# How Does the Valence of Wording Affect Features of a Scale? The Method Effects in the Undergraduate Learning Burnout Scale

**DOI:** 10.3389/fpsyg.2020.585179

**Published:** 2020-09-28

**Authors:** Biao Zeng, Hongbo Wen, Junjie Zhang

**Affiliations:** Collaborative Innovation Center of Assessment Toward Basic Education Quality, Beijing Normal University, Beijing, China

**Keywords:** learning burnout, valence of wording, method effect, parallel analysis, multitrait-multimethod model

## Abstract

A combination of both positively and negatively worded items is often employed in a survey to reduce participants’ acquiescence bias, but such a combination may hurt the validity of the survey. The current study investigated the effect of valence of wording on participants’ (*N* = 1132) responses to four versions of the Undergraduate Learning Burnout (ULB) scale. The results showed that the valence of wording affected a number of features of the scale. The internal consistency of both the original and the original-reverse versions (consisted of both positively and negatively worded items) was lower than that of the positive-only and the negative-only versions. The original and the original-reverse versions also had more factors than the positive-only and the negative-only versions. The original and the original-reverse versions showed method effects from both the positively and the negatively worded items, and those from the negatively worded items were stronger than those from the positively worded items. The method effects were predicted by participants’ subjective well-being and future academic career plans. Together, this study suggests that using a combination of positively and negatively worded items can lead to a predictable response style and significant method effects, which reduce the scale’s internal consistency and change the factor structure of the scale.

## Introduction

Likert scales are often used in developing surveys in the fields of psychology and education. These scales are consisted of a series of statements related to the target traits, evaluating the respondents’ attitudes, opinions, evaluations, and intentions regarding a specific object or event. However, people often show response bias or response set when responding to these scales. Response bias refers to a systematic tendency to respond to a questionnaire on some basis other than the specific item content (i.e., what the items were designed to measure; Cronbach, 1946; Paulhus, 1991). Often times, when responding to a statement on a Likert scale, people tend to take into account only one side of the statement while ignoring the other side. For example, one category of response bias is acquiescence bias, which refers to people’s tendency to select positively worded items rather than the negatively worded ones (Watson, 1992). In order to reduce the influence of response bias, researchers can use a combination of positively and negatively worded items in developing questionnaires. The negatively worded items can introduce cognitive “bumps” to the participants, which increases the chance for them to complete the survey more carefully (Podsakoff et al., 2003) and in turn increases both the scale’s accuracy in measuring the target characteristics (Anderson and Gerbing, 1988) and its measurement efficacy (Worcester and Burns, 1975; Chang, 1995).

An important assumption of using such a combination in formulating a scale is that both the positively and the negatively worded items should measure the same constructs (Marsh, 1996). However, research has shown that correlations between items within a scale that has both positively and negatively worded items are weaker than those within a scale that has only positively worded items (DiStefano and Motl, 2006; Carlson et al., 2011) and that using a combination of positively and negatively worded items reduces the internal consistency of a scale (Lee et al., 2008).

Using such a combination may also change the factor structure of the scale by bringing in unrelated factors to the target traits that the scale intends to measure (Corwyn, 2000; Vautier and Pohl, 2009; John et al., 2019), thereby violating the assumed unidimensional structure of the scale (Cordery and Sevastos, 1993; Jost and Thompson, 2000; Hevey et al., 2010). For example, Marsh et al. (2010) conducted confirmatory factor analysis (CFA) on the Rosenberg Self-Esteem Scale (RSES) and found that this unidimensional construct ended up having two additional factors, corresponding to the positively and negatively worded items, respectively, due to its mixed valence of wording. Some studies showed that in addition to the comprehensive self-esteem factor, either the positively worded items (Wang et al., 2001) or the negatively worded items (DiStefano and Motl, 2006) could bring a method factor. Other studies suggest that both the positively and the negatively worded items can induce method factors (Lindwall et al., 2012).

Such a change in the internal consistency and factor structure of a scale that has a mixed valence of wording can be attributed to the method effects. The method effects refer to tendencies to answer questions in survey-based criteria unrelated to the content being measured, which causes irrelevant systematic variance (American Educational Research Association, American Psychological Association, and National Council on Measurement in Education, 1999; Lindwall et al., 2012). Furthermore, both positively and negatively worded items can introduce significant method effects (Bolin and Dodder, 1990; Wang et al., 2001). Some studies demonstrate that the negatively worded items cause stronger method effects compared to positively worded items (Marsh, 1996; Quilty et al., 2006; DiStefano and Motl, 2009), whereas others show that it is the positively worded items that lead to the stronger method effects (Farh and Cheng, 1997; Lindwall et al., 2012). The differential direction in which valence of wording affects the strength of the method effects can be attributed to cultural differences. For example, the phenomenon that the positively worded items induce stronger method effects is particularly prevalent in China where people value modesty. As confirmed by Farh and Cheng (1997), Chinese people consistently underestimated their performance when responding to the RSES, resulting in stronger method effects being associated more with the positively worded items.

In order to explore the method effects on a scale’s validity, the multitrait-multimethod (MTMM) model in CFA is often employed to evaluate the discriminant and convergent validities (Campbell and Fiske, 1959). The MTMM model mainly includes two subtypes of specific models, the Correlated Trait-Correlated Method (CT-CM) and the Correlated Trait-Correlated Uniqueness (CT-CU). The CT-CM model treats method effects as a latent variable and separates the method variance from the characteristic variance. It is therefore able to measure the method effects directly from the method variance (Widaman, 1985). The CT-CU model, on the other hand, is proposed to solve the problem of low identification rate in the CT-CM model (Marsh, 1989). The CT-CU model classifies the method effects as residuals; however, this model does not assume independent method factors, which makes it unable to estimate the strength of the method effects directly or to examine the relationship between the method factors and external variables. For this reason, the CT-CM model is often used preferentially over other methods in estimating the method effects (Tomás and Oliver, 1999; Conway et al., 2004) unless it does not converge in practical applications (Lance et al., 2002; Wang, 2014). However, a drawback of the CT-CM model is that it allows for correlations among different method factors, which is not inevitable in actuality (Wang, 2014). In this case, the use of a more restricted Correlation Trait-Uncorrelated Method (CT-UM) model is more suitable (Widaman, 1985). Furthermore, many studies have found that the CT-UM model can better fit the data than other traditional models (Reise et al., 2007; Cao and Gu, 2010; Martel et al., 2010). Therefore, we opted for the CT-UM model to examine the method effects in this study.

Using a new research paradigm that can determine whether the latent factors of method effects are related to other constructs or variables, DiStefano and Motl (2009) confirm that the construct or variable of interest are related to the method effects, suggesting that the method effects are not simply a systematic error, but rather a stable style of responses related to the characteristics of the participants. In Quilty et al. (2006), participants who were emotionally stable were more likely to disagree with the negatively worded items, and participants with high avoidance motivation were more likely to agree with these items. Lindwall et al. (2012) found that participants with higher life satisfaction were more likely to agree with the positively worded items, whereas those with higher levels of depression were more likely to disagree with these items. In addition, participants’ current emotional valence can also predict the method effects. For example, in Brosan et al. (2011), participants with higher levels of anxiety experienced bias in information processing and were more susceptible to agree with the negatively worded items, resulting in a biased pattern in responses.

There are a number of limitations to previous research that discuss the effect of using a combination of positively and negatively worded items. First, previous research on the effects of valence of wording focuses almost exclusively on a single valence, yet the effect of using different combinations of both positively and negatively worded items remains underexplored. Second, there are mixed results of whether the valence of wording introduces significant method effects and of which valence introduces stronger method effects. Third, most research that investigated the method effects focused on the field of personality traits, but rarely tackled issues related to learning burnout. Learning burnout refers to students’ negative attitudes toward learning and dismissive behavior due to a lack of interest in learning or chronic tress related to learning (Lian et al., 2005). Learning burnout is an important construct to study due to its prevalence and adverse consequences, such as reduced enthusiasm and commitment to learning (Zhang et al., 2009), poor academic performance (Gao, 2013; Wang, 2019), lowered self-esteem (Shi and Tan, 2008), and an overall worse subjective well-being (Shan et al., 2010).

The goal of the current study is to investigate the effect of different valence of wording on the outcomes of the Undergraduate Learning Burnout (ULB) scale so as to estimate the parameters of the scale accurately and provide references for measuring the learning burnout in college students. We mainly focused on how valence of wording affected the internal consistency and factor structure of the ULB, whether there would be significant method effects, and which statements would be associated with stronger method effects. We also examined if students’ subjective well-being and future academic career plans would predict the method effects. We hypothesized that a combination of positively and negatively worded items would reduce the internal consistency, produce additional factors that are irrelevant to the variables of interest, and introduce significant method effects, with the negatively worded items inducing stronger method effects than the positively worded ones do. We also predicted that such method effects could be predicted by students’ subjective well-being and future academic career plans.

## Materials and Methods

### Participants and Materials

A total of 1132 students (368 males and 764 females) participated in this study. Among all participants, 414 were freshmen, 243 were sophomores, 66 were juniors, 170 were seniors, and 239 were graduate students.

The ULB scale used in this study was adopted by Lian et al. (2005) from the Marlach Burnout Inventory (Maslach and Jackson, 1981). The ULB scale has a high internal consistency as indicated by its overall *α* coefficient of 0.87. Lian et al. (2005) original ULB scale consisted of 20 items on five-point Likert scales: “1 = strongly disagree,” “2 = somewhat disagree,” “3 = uncertain,” “4 = somewhat agree,” and “5 = strongly disagree,” where higher scores indicated greater learning burnout. Importantly, in this study, we eliminated the “uncertain” option because research has shown that such an option may be chosen due to a number of factors irrelevant to the target traits of the participants (such as participants not understanding the questions or not having a clear viewpoint), inducing measurement error and threatening the scale’s reliability and validity (Raaijmakers et al., 2000; Kulas et al., 2008).

In addition to using the original ULB scale developed by Lian et al. (2005), we have also created three new versions of the scale by changing the valence of wording. The four versions of the ULB scale are therefore included: the original version (12 positively worded items and 8 negatively worded items), the original-reverse version (where we changed all positively worded items in the original version to negatively worded and vice versa), the positive version (20 positively worded items), and the negative version (20 negatively worded items). Additionally, a demographic survey and a two-item questionnaire querying participants’ subjective well-being and future academic career plans (whether they expected to pursue a master’s or doctoral degree) on four-point Likert scales similar to the ULB scale were also administered in all four conditions.

### Procedure

Participants completed the questionnaires online via a platform similar to the Amazon Mechanical Turk. Participants were allowed to choose one of the four versions of the ULB: 316 participants completed the original version, 267 did the original-reverse version, 288 did the positive version, and 261 did the negative version.

### Data Analysis

Data from 36 participants were excluded for outlying scores, which were defined as being more than 2.5 standard deviations away from the mean of total response scores (Liu, 2019). The final sample consisted of 1,096 participants (306 in original condition, 258 in original-reverse condition, 277 in positive condition, and 255 in negative condition).

We conducted a parallel analysis of scale dimensions on each of the four versions by comparing actual data points with 50 simulated random data points. If the variation explained by the factors in the actual data was even smaller than that in the simulated random data, then the factors were discarded (Reise et al., 2000). After determining the number of scale dimensions, we conducted exploratory factor analysis (EFA) on each of the four versions by building two models—which contained one factor and two factors, respectively—in Mplus 8.3. Because the scoring scales used in this study were four-point Likert scales, we have chosen the variation adjusted weighted least squares (WLSMV) estimation method to analyze our data. Research has shown that when the score range is no wider than four points, it is not appropriate to conduct analyses using the maximum likelihood estimation method because this method cannot give an accurate estimation of the parameter or the standard error (Finney and Distefano, 2006). In contrast, the WLSMV is designed specially to deal with category changes, and it performs better than other estimation methods when dealing with categorical data (Beauducel and Herzberg, 2006; Brown, 2006; Finney and Distefano, 2006). In addition, the WLSMV method can also obtain an accurate estimation of the parameter in both skewed and small sample data (Flora and Curran, 2004; Beauducel and Herzberg, 2006). The final EFA results were obtained after factor rotation via the GEOMIN oblique rotation method, which was to account for correlations and cross-loadings among factors.

**FIGURE 1:**
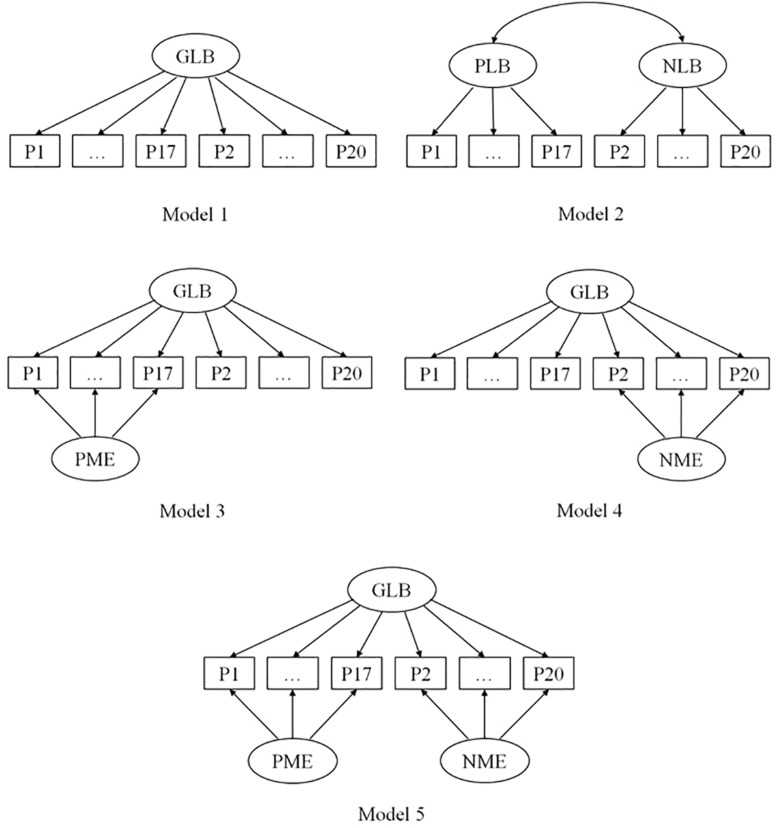
The five factor structure models of the original version of the ULB scale. GLB, general learning burnout factor; PLB, positive learning burnout factor; NLB, negative learning burnout factor; PME, positive method effect; NME, negative method effect.

CFA was also performed in Mplus 8.3 using the variation adjusted weighted least squares estimation method. A total of five models were established by adopting a method of the fixed variance of 1 (Figure 1). Two models, Model 1 and Model 2, were set up based on the results from the EFA. Three additional models were also set up for the original version and the original-reverse version, which might have method effects due to containing both positively and negatively worded items. Among these models, Model 1 contained only one substantive general learning burnout (GLB) factor. Model 2 contained two substantive factors of learning burnout, corresponding to positively and negatively worded items, respectively. Models 3, 4, and 5 adopted the CT-UM model to analyze the method effects. Model 3 and Model 4 both included a substantive learning burnout factor and a method factor. The method factor in Model 3 was obtained from all the positively worded items, and the method factor in Model 4 was obtained from all the negatively worded items. Model 5 contained one learning burnout factor and two method factors that were obtained from the positively and the negatively worded items, respectively.

Finally, a path analysis of the method factors was conducted in Mplus 8.3, using subjective well-being and academic career plans as the predictive variables and the latent method factor of the scale as the outcome variable, to explore whether the method effects could be predicted participants’ subjective well-being and academic career plans.

## Results

### Descriptive Statistics

Table 1 presents the mean response scores on each item in each of the four versions of the ULB scale. Scores on each item of the four versions were approximately normally distributed, as both the absolute values of the kurtosis and the skewness of all items were within 1. However, results from a Henze-Zirkler multivariate normality test suggested that none of the scores on all four versions showed normal distribution (*p* < 0.001; Korkmaz et al., 2014). The mean of score across all items in the positive version (*M* = 2.40, *SD* = 0.63) was the highest, followed by the original version (*M* = 2.35, *SD* = 0.75) and the original-reverse version (*M* = 2.34, *SD* = 0.71), and the negative version (*M* = 2.21, *SD* = 0.76) had the lowest scores. A one-way ANOVA showed that the difference in mean scores by version was significant, *F*(3,1092) = 12.22, *p* < 0.001. A Bonferroni *post hoc* test revealed that the mean score on the negative version was significantly lower than that on the original version (*M*_*diff*_ = −3.03, *p* < 0.001), the original-reverse version (*M*_*diff*_ = −2.51, *p* < 0.01), and the positive version (*M*_*diff*_ = −3.76, *p* < 0.01), whereas the mean scores on the latter three versions did not differ from each other.

**TABLE 1:** Descriptive statistics for the four versions of the ULB scale.

	Original		Original-Reverse		Positive		Negative	
				
	*M*	*SD*	*M*	*SD*	*M*	*SD*	*M*	*SD*
Item 1	2.37	0.73	2.24	0.76	2.30	0.59	2.13	0.74
Item 2	1.74	0.78	2.02	0.74	2.00	0.63	1.58	0.64
Item 3	2.45	0.70	2.28	0.69	2.49	0.61	2.16	0.72
Item 4	2.32	0.85	2.45	0.75	2.53	0.66	2.19	0.81
Item 5	2.51	0.77	2.41	0.69	2.47	0.61	2.43	0.78
Item 6	2.34	0.73	2.36	0.72	2.23	0.68	2.24	0.80
Item 7	2.60	0.79	2.83	0.71	2.71	0.59	2.48	0.77
Item 8	2.57	0.74	2.62	0.69	2.55	0.64	2.36	0.80
Item 9	2.12	0.72	2.51	0.66	2.57	0.62	2.07	0.72
Item 10	2.33	0.79	2.40	0.72	2.45	0.71	2.30	0.78
Item 11	2.25	0.67	2.04	0.70	2.28	0.60	1.92	0.66
Item 12	2.18	0.81	2.41	0.78	2.34	0.72	2.52	0.81
Item 13	2.25	0.78	2.10	0.81	2.18	0.64	1.92	0.74
Item 14	2.78	0.68	2.30	0.62	2.28	0.58	2.58	0.81
Item 15	2.25	0.74	1.77	0.77	2.28	0.65	1.77	0.69
Item 16	2.29	0.82	2.38	0.71	2.47	0.67	2.16	0.78
Item 17	2.41	0.66	2.36	0.64	2.47	0.62	2.29	0.72
Item 18	2.51	0.73	2.24	0.65	2.39	0.60	2.36	0.79
Item 19	2.37	0.78	2.24	0.66	2.34	0.61	2.27	0.78
Item 20	2.59	0.78	2.74	0.76	2.60	0.66	2.45	0.84

*Original, original version of the ULB scale; Original-Reverse, original-reverse version of the ULB scale; Positive, positive version of the ULB scale; Negative, negative version of the ULB scale.*

### Internal Consistency Analysis

The internal consistency of both the original (Cronbach’s alpha = 0.79) and the original-reserve (Cronbach’s alpha = 0.81) versions was lower than that of both the positive (Cronbach’s alpha = 0.91) and the negative versions (Cronbach’s alpha = 0.91).

### Exploratory Factor Analysis

**FIGURE 2:**
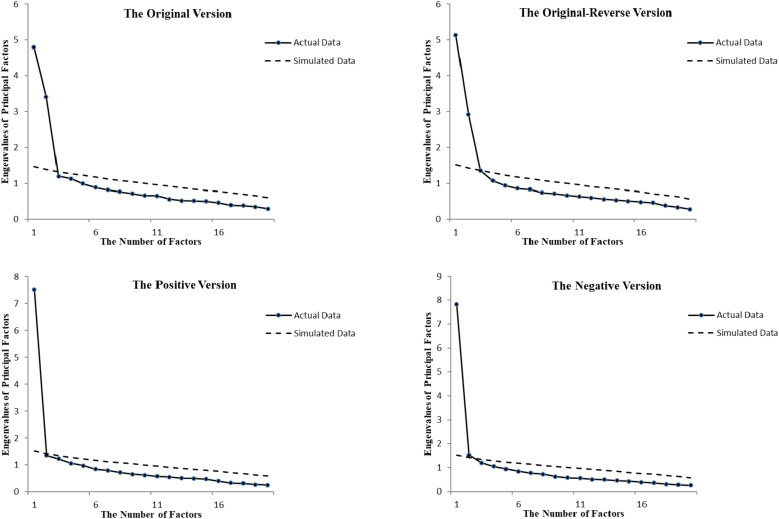
The solid lines represent scree plot obtained from the actual data. The dashed lines are scree plot averaged across 50 data points in a simulated dataset generated from a population, where all variables are uncorrelated, with the same number of participants and items as the original dataset.

Results from the Kaiser–Meyer–Olkin Sampling Adequacy Measure and Bartlett’s spherical test have confirmed that our data were appropriate for factor analysis. Results from a parallel analysis showed that both the original and the original-reverse versions had more factors than the positive and negative versions (Figure 2). Specifically, the original and original-reverse versions had two factors because two eigenvalues were much higher than the average eigenvalues of the random matrix, whereas the positive and the negative versions each had only one factor because only one eigenvalue was much higher than the average eigenvalue of the random matrix for each version. Although in the negative version, the eigenvalue of the second factor was also above the average eigenvalue curve of the random matrix, the difference was neglectable as it might be due to sampling or other random errors.

We then created two models, one with one factor (Model 1) and the other with two factors (Model 2), based on the results from the parallel analysis and conducted EFA using these models on each version of the ULB scale. Tables 2, 3 present the factor loading of each of the four versions that we obtained. Under Model 1, the factor loading of the positive (*M* = 0.66) and the negative (*M* = 0.63) versions was higher than that of the original (*M* = 0.35) and the original-reverse versions (*M* = 0.45). Furthermore, the factor loading of most items in the positive and the negative versions was above 0.50, indicating that these two versions were in line with the one-factor structure; on the other hand, the factor loading of many items in the original and the original-reverse versions was lower than 0.30, with that of some even being negative values, suggesting that these versions might not fit into the one-factor structure. Under Model 2, the correlation between the two factors was much weaker in the original (*r* = −0.08, *p* > 0.05) and the original-reverse versions (*r* = 0.13, *p* > 0.05) than in the positive (*r* = 0.64, *p* < 0.05) and the negative versions (*r* = 0.71, *p* < 0.05). In addition, the positive and the negative versions showed cross-factor loading in many items, indicating that these two versions were well-suited for the one-factor structure. In contrast, most items in the original and the original-reverse versions had a higher loading on one factor and a lower loading on the other one, with only a few items showing cross-factor loading, suggesting that these two versions were better suited for the two-factor structure rather than the one-factor structure.

**TABLE 2:** Standardized factor loadings for the four versions of the ULB scale in Model 1.

	Original	Original-Reverse	Positive	Negative
Item 1	0.05	0.28*	0.62*	0.63*
Item 2	0.46*	0.54*	0.50*	0.47*
Item 3	−0.14*	0.16*	0.68*	0.71*
Item 4	0.66*	0.74*	0.64*	0.67*
Item 5	0.77*	0.83*	0.81*	0.82*
Item 6	−0.12*	−0.01*	0.57*	0.63*
Item 7	0.50*	0.52	0.67*	0.61*
Item 8	−0.14*	−0.11	0.53*	0.63*
Item 9	0.68*	0.71*	0.63*	0.77*
Item 10	0.61*	0.66*	0.70*	0.68*
Item 11	−0.11	0.20*	0.69*	0.69*
Item 12	0.54*	0.52*	0.54*	−0.01
Item 13	0.01	0.17*	0.65*	0.65*
Item 14	0.60*	0.76*	0.79*	0.65*
Item 15	−0.13*	0.16*	0.58*	0.65*
Item 16	0.67	0.67*	0.71*	0.65*
Item 17	−0.01	0.40*	0.82*	0.65*
Item 18	0.75*	0.74*	0.81*	0.65*
Item 19	0.71*	0.73*	0.69*	0.65*
Item 20	0.69*	0.41*	0.58*	0.66*

*p < 0.05.

**TABLE 3:** Standardized factor loadings for the four versions of the ULB scale in Model 2.

	Original		Original-Reverse		Positive		Negative	
				
	Factor 1	Factor 2	Factor 1	Factor 2	Factor 1	Factor 2	Factor 1	Factor 2
Item 1	0.17	0.67*	0.19	0.50*	0.15	0.55*	0.00	0.71*
Item 2	0.48*	0.12	**0**.53*	0.06	**0**.47*	0.07	0.00	0.54*
Item 3	−0.03	**0**.67*	−0.01	**0**.68*	**0**.58*	0.16	**0**.50*	0.27
Item 4	**0**.66*	0.02	**0**.74*	0.02	**0**.65*	0.03	**0**.76*	−0.07
Item 5	**0**.78*	0.04	**0**.83*	0.01	***0***.56*	*0*.*34*	**0**.68*	0.19
Item 6	−0.03	**0**.53*	−0.10	**0**.40*	**0**.43*	0.20	**0**.73*	−0.08
Item 7	**0**.47*	−0.24	**0**.58*	−0.27	0.74*	−0.03	0.88*	−0.29
Item 8	−0.05	0.62*	−0.21	0.39*	0.42*	0.16	0.67*	−0.02
Item 9	0.71*	0.12	0.72*	−0.07	0.76*	−0.10	0.71*	0.10
Item 10	0.63*	0.12	0.67*	−0.06	−0.10	0.89*	0.04	0.73*
Item 11	0.00	0.68*	0.03	0.69*	*0*.*32*	0.46*	0.27	0.50*
Item 12	0.53*	−0.04	0.55*	−0.12	0.32*	0.28	0.23	−0.27*
Item 13	0.10	0.57*	0.04	0.55*	0.44*	0.28	*0*.*34*	***0***.37*
Item 14	0.59*	−0.05	0.76*	0.01	0.51*	*0.37*	0.37*	0.02
Item 15	−0.01	0.66*	−0.03	0.77*	0.14	0.51*	0.14	**0.43**
Item 16	0.67*	0.00	0.68*	−0.05	0.07	0.72*	−0.04	0.75*
Item 17	0.11	0.69*	*0.31*	0.54*	0.90*	−0.03	0.74*	0.08
Item 18	0.75*	−0.01	0.74*	0.01	0.72*	0.15	0.71*	0.13
Item 19	0.70*	−0.05	0.71*	0.07	0.00	0.78*	0.04	0.81*
Item 20	0.67*	−0.14	0.42*	−0.04	*0*.*30*	0.35*	0.61*	0.09

*The higher factor loadings for each item within one version of the ULB were expressed in bold, and the factor with cross-loadings (both factor loadings being greater than 0.30) were italicized.*

### Confirmatory Factor Analysis

We conduct CFA using both Model 1 and Model 2 on the EFA results from each version of the ULB (Table 4). The comparative fit index (CFI) value for the ideal model fit should be no less than 0.90 (Hu and Bentler, 1999). The root mean square error of approximation (RMSEA) value should be no greater than 0.08 (Browne and Cudeck, 1992), with the highest acceptable range being no greater than 0.10 (Steiger, 1990). Under Model 1, the CFI values of the original and the original-reverse versions were 0.27 and 0.64, and the RMSEA values were 0.19 and 0.14, respectively, all of which indicated that these versions did not fit into the one-factor model and were therefore not composed of a unidimensional structure. On the other hand, the CFI values of the positive and negative versions were 0.93 and 0.93, and the RMSEA values were 0.09 and 0.10, respectively. Although the RMSEA values of these two version scales still did not reach the ideal level, they were in the acceptable range, suggesting that these two versions fitted in the one-factor model as they had only one learning burnout factor. Under Model 2, the positive and negative versions, the CFI values were 0.95 and 0.96, and the RMSEA values were 0.08 and 0.07, respectively. However, as these two versions fitted in the one-factor model ideally, dividing their factor structure into two dimensions would not have much practical significance. As for the original and original-reverse versions, the CFI values were 0.86 and 0.84, and the RMSEA values were 0.08 and 0.09, respectively. The level of model fitting improved greatly but still has not reached the ideal fit. These results suggest that these two versions are not a simple combination of two learning burnout factors, but rather, it is possible that multi-dimensional structure of the original and the original-reverse versions is a result of having both one learning burnout factor plus one or two other factors induced by method effects.

**TABLE 4:** Model fit indices for in the four versions of the ULB scale in Model 1 and Model 2.

	χ^2^	*df*	CFI	RMSEA [90% CI]
**Model 1**				
Original	1954.91	170	0.27	0.19 [0.18, 0.19]
Original-Reverse	1030.15	170	0.64	0.14 [0.13, 0.15]
Positive	580.44	170	0.93	0.09 [0.09, 0.10]
Negative	559.48	170	0.93	0.10 [0.09, 0.10]
**Model 2**				
Original	504.41	169	0.86	0.08 [0.07, 0.09]
Original-Reverse	545.67	169	0.84	0.09 [0.08, 0.10]
Positive	467.57	151	0.95	0.09 [0.08, 0.10]
Negative	364.53	151	0.96	0.07 [0.07, 0.08]

To test the aforementioned idea, we created Models 3, 4, and 5 by adding one positive, one negative, or both method factors to Model 1. The CFA results of the original and the original-reverse versions based on these three models are shown in Table 5. For the original version, the level of fitting in Model 3 was slightly worse than that in Model 2, with a CFI value of 0.86 and an RMSEA value of 0.08. The level of fitting of the original-reverse version in Model 3 was better than that in Model 2, with a CFI value of 0.85 and an RMSEA value of 0.09. The level of fitting in Model 4 for both versions was better than that in both Model 2 and Model 3, with the CFI values being 0.89 for the original version and 0.89 for the original-reverse version, and the RMSEA values being 0.08 and 0.08, which were close to the ideal fit. Model 5 was the most ideal among all models created. The CFI values were 0.90 and 0.92, and RMSEA values were 0.08 and 0.07, for the two versions, respectively. These results suggest versions of the ULB that contained both positively and negatively worded items had one learning burnout factor and two additional method factors for the positively and negatively worded items, respectively.

**TABLE 5:** Model fit indices for the original and the original-reverse versions of the ULB scale in Models 3, 4, and 5.

	χ^2^	*df*	CFI	RMSEA [90% CI]
**Original**				
Model 3	507.31	162	0.86	0.08 [0.08, 0.09]
Model 4	435.96	158	0.89	0.08 [0.07, 0.08]
Model 5	406.50	150	0.90	0.08 [0.07, 0.08]
**Original-Reverse**				
Model 3	510.95	158	0.85	0.09 [0.08, 0.10]
Model 4	434.67	162	0.89	0.08 [0.07, 0.09]
Model 5	349.83	150	0.92	0.07 [0.06, 0.08]

In order to further explore the method effects in the original and original-reverse versions, we analyzed the loading of the method factors in Model 5 (Table 6). The results showed that the loading of the negative method factor (original: *M* = 0.62; original-reverse: *M* = 0.56) was higher than that of the positive one (original: *M* = 0.01; original-reverse: *M* = 0.03) for both versions, with the loading of all negatively worded items reaching significance (*p* < 0.05) except for one insignificant case. As for the positively worded items, many of those items in the original version bared a factor loading of a negative value, and only one item’s factor loading was significant (*p* < 0.05). These results suggest that versions of the ULB scales that contained both positively and negatively worded items had more method effects from the negatively worded items than from the positively worded items.

**TABLE 6:** Standardized factor loadings for each item of the original and the original-reverse versions of the ULB scale in Model 5.

	Original		Original-Reverse	
		
	PME	NME	PME	NME
Item 2		0.47*	0.28*	
Item 4		0.66*	0.45*	
Item 5		0.77*	0.35*	
Item 7		0.49*	0.54*	
Item 9		0.70*	0.47*	
Item 10		0.63*	0.00	
Item 12		0.53*	0.26*	
Item 14		0.60*	0.40*	
Item 16		0.67*	0.06	
Item 18		0.75*	0.30*	
Item 19		0.70*	-0.08	
Item 20		0.68*	0.52*	
Item 1	−0.22			0.47*
Item 3	0.08			0.69*
Item 6	−0.15			0.40
Item 8	−0.23			0.40*
Item 11	0.23			0.69*
Item 13	−0.07			0.55*
Item 15	0.55*			0.75*
Item 17	−0.11			0.57*

PME, method effect induced by positively worded items; NME, method effect induced by negatively worded items. *p < 0.05.

### Correlates of Method Effects

We conducted path analysis to explore whether some other variables, such as the subjective well-being and future academic career plans, can predict the method effects. We took these two variables as the predictor variables and the two potential method factors of the original version of the ULB (as outlined in Model 5) as the outcome variables. As presented in Table 7, we found a strong negative correlation between future academic career plans and the learning burnout factor, *r* = −0.62, *p* < 0.001, but there was no significant correlation between subjective well-being and learning burnout factor, *r* = 0.28, *p* > 0.05. However, there was a strong negative correlation between subjective well-being and the method factor induced by the positively worded items, *r* = −0.86, *p* < 0.001, and a strong positive correlation between subjective well-being and the method factor induced by the negatively worded items, *r* = 0.50, *p* < 0.001, suggesting that participants with higher subjective well-being were more likely to agree with the negatively worded items and to disagree with the positively worded items. On the contrary, there was a strong positive correlation between future academic career plans and the method factor induced by the positively worded items, *r* = 0.65, *p* < 0.001, and a strong negative correlation between future academic career plans and the method factor induced by the negatively worded items *r* = −0.78, *p* < 0.001, suggesting that participants with higher future academic career plans were more likely to agree with the positively worded items and to disagree with the negatively worded items. In addition, both subjective well-being and future academic career plans were negatively correlated with learning burnout scores, *r* = −0.25, *p* < 0.01; *r* = −0.21, *p* < 0.01, suggesting that the higher one’s subjective well-being or future academic career plans are, the less learning burnout they have.

**TABLE 7:** Correlations between latent factors of the original ULB scale and their two potential predictors (subjective well-being and future academic career plans).

	GLB	PME	NME
Subjective well-being	0.28	−0.86***	0.50***
Future academic career plans	−0.62**	0.65***	−0.78***

**p < 0.01, ***p < 0.001.

## Discussion

In this study, we explored the impact of the valence of wording on the scale measurement through four versions of the ULB scale with different combinations of positively and negatively worded items. Using new statistical methods and modeling, we confirmed that a combination of both positively and negatively worded items undermines the internal consistency and changed the factor structure of the scale by introducing additional method factors. More specifically, we showed that both positively and negatively worded items can induce method effects in scales that contain both positively and negatively worded items and that such method effects can be predicted by participants’ subjective well-being and future academic career plans.

Our results confirmed that mixed valence of wording (or using a combination of positively and negatively worded items) reduces the scale’s internal consistency as a result of the method effects, which was consistent with the results in previous literature (Lee et al., 2008). Both versions with only unidirectional wording had greater internal consistency than both with a combination of positive and negative wording. Because the contents of the four versions were the same, differences in internal consistency can only be explained by the effect of the wording valance. We therefore suggest that the contents measured by different valences of wording are actually not completely consistent as the combination of different valences of wording introduces irrelevant variations to the scale (DiStefano and Motl, 2006; Lee et al., 2008; Carlson et al., 2011). Specifically speaking, the original and original-reverse version scale included both positively and negatively worded items, negatively worded items were more complicated than positively worded items, and the information processing methods required by the equivalent negatively and positively worded items were not inconsistent (Mayo et al., 2004), which made participants’ responses to positively and negatively worded items different. As a result, the correlation between the scores of different items in the scale decreased, eventually causing a decrease in the internal consistency of the scales.

Our parallel analysis and EFA showed that a scale with a unidirectional valence of wording has a unidimensional structure, whereas one with a mixed valence of wording changes such a structure by bringing additional factors. These findings are consistent with previous literature, where two factors are present even if only one construct was measured when a scale employs mixed valence of wording (Conroy, 2004; DiStefano and Motl, 2009). A commonly agreed explanation for such a structural change is the method effects induced by mixed valences of wording. However, there is still controversy regarding which factors are responsible for the multidimensional structures and whether the positively and negatively worded items measure the same traits. Some researchers argue that positively and negatively worded items measure different traits (Joireman et al., 2008; Boduszek et al., 2013), while others suggest that those items measure the same trait and that the multidimensional structures are only a result of method effects induced by different valences of wording. For example, Wang et al. (2010) and Cai (2017) suggest that the multidimensional structure of the RSES is composed of a self-esteem factor and a method factor. The results of the current study support the latter argument. Our data fit in models that contained method factor(s) better than in the model that did not; more importantly, the model that contained a learning burnout factor and two method factors (for positively and negatively worded items, respectively) fitted the data best, suggesting that the two-factor structure of the ULB scale is composed of a learning burnout factor and two method factors.

However, our findings that the ULB scale contained only one substantive learning burnout factor is inconsistent with the three-factor structure that was originally assumed. It is likely due to the use of different criteria in determining the number of factors in the EFA. Based on Kaiser’s rule, which supports the three-factor structure, whether a factor will be retained is determined by whether the eigenvalues of the actual data are greater than one (Henson and Robert, 2006). Although Kaiser’s rule was one of the most commonly used criteria, it tends to retain too many factors (Zwick and Velicer, 1986). In contrast, the method of parallel analysis use in the current study is considered the most precise method for retaining factors and is better than many other methods, including Kaiser’s rule (Bartlett, 1950; Humphreys and Montanelli, 1975; Zwick and Velicer, 1986; Silverstein, 1987). The parallel analysis determines whether a factor is retained based on comparisons with the real data by comparing the eigenvalue against the average eigenvalue of the random matrix. Our findings indicate that the traditional Kaiser’s rule may overestimate the number of factors, whereas using parallel analysis can avoid this problem and give a more accurate estimate of how many factors a scale has.

A further analysis of loading on the method factors in the model that contains a learning burnout factor and two method factors (Model 5) for the two mixed-valence versions shows that the method effect induced by the negatively worded items is stronger than that by the positively worded items. These findings are consistent with many previous studies (Marsh, 1996; Quilty et al., 2006; DiStefano and Motl, 2009). We propose three possible explanations for this phenomenon. The first is related to the negatively worded items themselves: The negative expression of a statement usually requires more complex cognitive processing and therefore pose difficulties in semantic understanding, which, in turn, affects the participants’ answers and leads to a greater measurement bias (Podsakoff et al., 2003). This explanation is supported by the finding that the mean score of the negative version was lower compared to the other three versions, and such a tendency was present in only the negative version but not in the positive version (as scores on the positive version did not differ from those of the two combination versions), suggesting that participants’ responses are more likely to be affected by negatively worded items than by positively worded items. The second explanation is related to the age and traits of the participants: previous research has shown that young people are more susceptible to negative information than older people (Carstensen and Mikels, 2005). Participants in the current study are all college students; they are therefore more likely to agree to the negatively worded items, which might in turn induce a stronger negative method effect. The third explanation is related to cultural differences. Culture plays a role in the strength of method effects. For example, Chinese people, living in a collectivist culture and valuing modesty, usually report a lower level of self-esteem in an attempt to maintain social approval, resulting in a stronger method effect in positively worded items (Farh and Cheng, 1997). However, this phenomenon is domain-specific, and it is more likely to present when assessing positive traits (such as self-esteem) or self-evaluating one’s own performance (Schmitt and Allik, 2005). Namely, not all traits will show this tendency, with learning burnout being one of the cases. Due to China’s strong competitive culture and huge employment pressure, Chinese students generally have a higher level of learning burnout than students in other countries (Jacobs and Dodd, 2003; Sun, 2019). This is the opposite of the case of self-esteem, and therefore, there is a stronger method effect in the negatively worded items.

The tendency for participants with high subjective well-being to agree with the negatively worded items and disagree with the positively worded items and the reversed tendency for participants with high academic expectations confirms that the method effects represent a stable response style and can be predicted by positive emotional traits (DiStefano and Motl, 2009). Yet, our findings on the directions of the correlations are somewhat surprising. Previous studies have shown that people with positive emotions are more likely to agree with positively worded items, while those with negative emotions are more likely to recognize negatively worded items (Podsakoff et al., 2003; Lindwall et al., 2012). In the current study, however, only the academic expectation measure is consistent with this tendency. The seemingly counterintuitive findings regarding subjective well-being, which is also a positive emotion, may be explained by the desensitization theory. Desensitization refers to the phenomenon in which individuals’ cognitive, emotional, physiological, and behavioral responses to a stimulus are reduced or eliminated over time due to repeated exposure (Funk, 2005). Individuals with higher subjective well-being are more likely to experience or pay attention to positive stimuli in general, which makes their cognitive and emotional responses to such stimuli gradually decline over time; yet, stimuli containing negative words are relatively rare or less attended to, leading to higher sensitivity to these stimuli. This phenomenon therefore still supports the nature of method effects as a stable response style of participants.

We should take measures to reduce the method effect on the measurement as much as possible. This can be achieved through two methods. The first is program control, or changing the valence of wording. More specifically, we can convert unipolar items into bipolar items. In other words, we can change a unidirectional item into one that contains a pair of antonyms (Schweizer and Schreiner, 2010). For instance, the statement that “mastery of professional knowledge is easy for me” can be changed to “I feel mastery of professional knowledge” with two options—“easy” and “difficult”—for participants to choose from. The second method is statistical control. As demonstrated by the current study, we can use MTMM models (such as the CT-UM model) to test for method effects. Such models are especially helpful when the target scale contains both positively and negatively worded items. If the target scale has significant method effects, then the method factors should be included in the model when performing CFA so that the parameters of the scale can be corrected, and the actual parameters can be accurately estimated.

One limitation of the current study is the lack of random assignment—participants were free to choose to answer one of the four versions of the ULB scale. However, note that the participants were blind to the conditions that they were asked to choose from (they were given four numbers to choose from without any other labeling), nor did they know how the four versions differed until they were debriefed after completing the study. Another limitation is that the sample composition was not well-balanced as our sample consisted of predominantly females (65%), which may hurt the generalizability of our results to the entire population. Future research should conduct large-scale sampling and exercise more control over the assignment of conditions to obtain even more convincing and reliable results. In addition, even after the negatively worded items in the original scale were converted to positively worded items (for the original-reverse version), some of these items still had a significant factor loading on method effects, which may be due to some random factors unrelated to the measurement. This study was not designed to separate these unrelated factors; future research can analyze the potential factors involved in this phenomenon. Finally, the current study employs an anchor test as it used the four different versions of the ULB scale; another future research direction is to conduct an anchor test analysis to further investigate method effects.

## Data Availability Statement

The raw data supporting the conclusions of this article will be made available by the authors, without undue reservation.

## Ethics Statement

Ethical review and approval was not required for the study on human participants in accordance with the local legislation and institutional requirements. The patients/participants provided their written informed consent to participate in this study.

## Author Contributions

BZ contributed to developing the research design, collecting data, conducting data analyses, and writing up the research in Chinese and revising the translated manuscript. HW, similarly, contributed to guiding the research design and revising the manuscript both before and after it was translated into English. JZ contributed to revising and improving the write-up. All authors contributed to the article and approved the submitted version.

## Conflict of Interest

The authors declare that the research was conducted in the absence of any commercial or financial relationships that could be construed as a potential conflict of interest.
